# LncRNA-MALAT1 promotes neovascularization in diabetic retinopathy through regulating miR-125b/VE-cadherin axis

**DOI:** 10.1042/BSR20181469

**Published:** 2019-05-15

**Authors:** Ping Liu, Song-Bai Jia, Jing-Ming Shi, Wen-Jie Li, Luo-Sheng Tang, Xia-Hua Zhu, Ping Tong

**Affiliations:** 1 Department of Ophthalmology, The Second Xiangya Hospital of Central South University, Changsha 410011, P.R. China; 2 Hunan Clinical Research Center of Ophthalmic Disease, Changsha 410011, P.R. China; 3 Department of Ophthalmology, The Third Xiangya Hospital of Central South University, Changsha 410013, P.R. China

**Keywords:** Diabetic retinopathy, MALAT1, miR-125b, neovascularization, VE-cadherin/β-catenin complex

## Abstract

**Background:** Diabetic retinopathy (DR) is currently the leading cause of blindness and visual disability in adults with diabetes mellitus (DM). Neovascularization has been identified as an important clinical property in DR, however, the exact mechanisms in DR neovascularization are still unclear and need further elucidation.

**Methods:** Quantitative real-time PCR (qRT-PCR) was conducted to detect the expression level of long non-coding RNA (lncRNA)-metastasis associated lung adenocarcinoma transcript 1 (MALAT1), miR-125b and vascular endothelial-cadherin (VE-cadherin) in human retina microvascular endothelial cells (hRMECs) treated with high glucose (HG). Luciferase assay was used to detect interaction of MALAT1 with miR-125b and miR-125b with VE-cadherin. MTT assay, transwell assay, tube formation assay and vascular permeability assay were conducted to detect the cell viability, migration tube formation ability and permeability of hRMECs, respectively. ELISA was used to examine the release of VE-cadherin and vascular endothelial growth factor (VEGF). Western blotting was used to access the protein expression of VE-cadherin, VEGF, β-catenin, matrix metalloproteinase (MMP) 2 (MMP2) and MMP9.

**Results:** MALAT1 and VE-cadherin were up-regulated while miR-125b was down-regulated in hRMECs treated with HG. MALAT1 could competitively bind to miR-125b against VE-cadherin at the site of 3′-untranslated region (3′-UTR), leading to the up-regulation of VE-cadherin. Knockdown of MALAT1 inhibited the proliferation, migration, tube formation and vascular permeability of hRMECs induced by HG through up-regulating miR-125b. Furthermore, we found the deletion of MALAT1 suppressed the VE-cadherin/β-catenin complex and neovascularization related proteins expression, which was up-regulated by HG.

**Conclusion:** Knockdown of MALAT1 inhibited cell proliferation, migration and angiogenesis of hRMECs via suppressing the VE-cadherin/β-catenin complex through targeting miR-125b. Inhibition of MALAT1 may serve as a potential target for anti-angiogenic therapy for DR.

## Introduction

Diabetic retinopathy (DR) is a frequent microvascular complication of patients with diabetes mellitus (DM) and one of the most common sight-threatening conditions, eventually leading to severe vitreous cavity bleeding, retinal detachment and vision loss [1]. The clinical symptoms of DR have been mainly identified as hemorrhages, lipid exudates and neovascularization [2,3]. Therefore, retinal microvascular pathology is important for understanding retinal degenerations during DR. For a longer period of time, the typical treatment choice of DR neovascularization with laser photocoagulation does not have a significant improvement in visual acuity. In order to reduce the limitations of current treatment options, it is urgent to explore the mechanism of DR neovascularization, and assess novel pharmacological therapies to target the essential biochemical mechanisms that promote DR.

The severity of diabetes plays a key role in DR, and several genes have been reported to be relevant with DR neovascularization [4]. Among these markers, vascular endothelial-cadherin (VE-cadherin) stands out as a reliable biomarker of angiogenesis activity [5]. VE-cadherin is a cell adhesion molecule localized at the endothelial junction, and plays an important role in angiogenesis and vascular permeability [6]. Recent studies suggested that the expression of VE-cadherin in the retina of diabetics was significantly increased compared with non-diabetic retinas [7]. Moreover, high glucose (HG) could increase VE-cadherin expression in human retina microvascular endothelial cells (hRMECs) [8]. However, the molecular mechanism of regulating VE-cadherin is not yet clear.

Long non-coding RNAs (lncRNAs) are identified as the transcripts >200 nucleotides and involved in various biological processes, such as cell proliferation, migration, cell cycle, cell apoptosis and angiogenesis [9]. Studies have found that lncRNA-metastasis associated lung adenocarcinoma transcript 1 (MALAT1) is associated with DM [10]. Recently, evidences indicated that MALAT1 could induce retinal microvascular dysfunction in diabetics [11]. Moreover, MALAT1 knockdown significantly alleviated diabetes-induced microvascular dysfunction *in vivo* and inhibited endothelial cell migration, tube formation and proliferation *in vitro* [11]. However, the underlying mechanism of action of MALAT1 in DR remains unclear.

MicroRNAs (miRNAs) are short non-coding RNAs of approximately 22 nucleotides in length [12]. miRNAs regulate gene expression through transcriptional or post-transcriptional regulation, inducing mRNA destabilization or inhibiting protein translation by binding to the seed region in the 3′-untranslated region (3′-UTR) of target genes [13]. miR-125b is a very active member of the miRNA family and down-regulated in DR [14]. Emerging evidences suggested that knockdown of miR-125b was correlated with the up-regulation of target genes, leading to tube formation in endothelial cells and DR *in vitro*. Chang and Hu [15] reported that overexpression of MALAT1 could repress the tumor inhibitory effect of miR-125b mimics. Furthermore, miR-125b was recently reported to inhibit tube formation of blood vessels through translational suppression of VE-cadherin in endothelial cells [16]. Thus, we speculated that MALAT1 might promote angiogenesis of retinal vascular endothelial cells induced by HG through targeting miR-125b and then promoting the expression of VE-cadherin.

## Materials and methods

### Cell culture and treatment

hRMECs were purchased from American Type Culture Collection (ATCC, U.S.A.). hRMECs were cultured in endothelial cell medium (ECM, Gibco) supplemented with 10% fetal bovine serum (FBS, Gibco, U.S.A.), penicillin (100 U/ml) and streptomycin (100 U/ml) at 37°C under 5% CO_2_. To detect the effect of glucose on hRMECs, the medium was further supplemented with high concentration of glucose (25 mM; HG) or high concentration of mannose (5.5 mM glucose and 19.5 mM mannose; OS).

### Cell transfection

MALAT1-specific short hairpin RNAs (shRNA) were cloned into pSicoR vector (shMALAT1). miR-125b mimics and miR-125b inhibitor were purchased from GenePharma (Shanghai, China). Upon reaching approximately 70–80% confluence, cells were transfected with miR-125b mimics, miR-125b inhibitor or shMALAT1 using Lipofectamine 2000 (Invitrogen, U.S.A.) according to the manufacturer’s instructions.

### Luciferase reporter assay

The procedure for the pMIR-luciferase reporter assay has been previously reported by Muramatsu et al. [16]. HRMECs were planted in 96-well plates at 5 × 10^3^ cells per well. Lipofectamine 2000 (Invitrogen, U.S.A.) was used to perform co-transfection of pMIR-luciferase reporter construct (Addwild type or mutant MALAT1 and VE-cadherin 3′-UTR), pRL-TK *Renilla*, miR-125b inhibitor or miR-125b mimics. After 24-h incubation, pMIR-luciferase activity was detected by Dual-luciferase Reporter Assay System (Promega, U.S.A.) as described in the manufacturer’s instructions.

### MTT assay

To prepare MTT solution, 5 mg MTT was dissolved in 1 ml PBS. After transfection and HG treatments, cells in 96-well plate were added with 10 μl MTT solution per well and cultured at 37°C for 4 h. Then the supernatant was discarded and the wells were washed by PBS. Each well was added with 100 μl DMSO, and the MTT formazan crystals were dissolved. The absorbance was measured at 570 nm using an ELX800 UV universal microplate reader (Bio-Tek Instruments Inc., Vermont, U.S.A.).

### Transwell assay

Transwell assay of cell motility was conducted as previously reported by Zhang et al. [17]. hRMECs were equally seeded in upper chamber (2.5 × 10^3^ cells/well) using culture medium without serum. In the lower chamber, 500 μl culture medium with 10% FBS was added. After 24 h cultivation, hRMECs in the upper chamber were removed using cotton swabs. hRMECs moved to lower chambers were stained by 0.1% Crystal Violet, and photographed by a LEICA DMI 4000B microscope. The number of cells in five randomly selected in fields was counted.

### Tube formation assay

Tube formation assay was performed as previously reported [2]. The basement membrane matrix (BMM) was placed into each well and then hardened for 30 min at 37°C. hRMECs (2 × 10^5^ cells per well) were seeded on the top of BMM-coated wells and then cultured for 24 h at 37°C. Images were obtained using Olympus IX70 microscope (Toyko, Japan).

### Measurement of vascular permeability

Measurement of vascular permeability was conducted as previously reported [2]. hRMECs were cultured with Evans Blue dye for 2 h and cleared the dye by PBS. Then Evans Blue dye was extracted by formamide for 18 h at 70°C. The extract was centrifuged at 10000×***g*** for 30 min at 4°C. The absorbance of the supernatant was measured at 620 nm. The concentration of Evans Blue was calculated from a standard curve and normalized to the dry weight of retina.

### ELISA

The release of vascular endothelial growth factor (VEGF) and VE-cadherin was detected by the ELISA kit (Roche, Indianapolis, IN) according to the manufacturer’s instructions. The supernatant of hRMECs was subjected to VEGF and VE-cadherin ELISA.

### RNA extraction and quantitative real-time PCR

Total RNA was extracted using TRIzol reagent (Invitrogen, U.S.A.), and conversed to cDNA using Superscript III reverse transcriptase (Invitrogen, U.S.A.). Quantitative real-time PCR (qRT-PCR) was performed with qRT-PCR Kits (Invitrogen, U.S.A.) according to the manufacturer’s instructions and an ABI 7300 Sequencing Detection System (Applied Biosystems, Foster City, CA, U.S.A.). The amplification conditions were as follows: initial denaturation at 95°C for 10 min, followed by 35 cycles of 10 s at 95°C, 15 s at 60°C, and 10 s at 72°C. The relative expression level of miRNA and mRNA was normalized to U6 small nuclear RNA and β-actin, respectively. The following primers were used for analysis: MALAT1 forward: 5′-AGGTAAAGCTTGAGAAGAT-3′, reverse: 5′-GGAAGTAATTCAAGATCAA-3′; miR-125b forward: 5′-CCAGATAC TGCGTATGTGTG-3′, reverse: 5′-GTCACCTGATCCCATCTAAC-3′; VE-cadherin forward: 5′-ATCGGTTGTTCAATGCGTCC-3′, reverse: 5′-CCTTCAGGATTTGGT ACATGACA-3′; U6 forward: 5′-CTCGCTTCGGCAGCACA-3′, reverse: 5′-AACG CTTCACGAATTTGCGT-3′; β-actin forward: 5′-AAATCTGGCACCACACCTT C-3′, reverse: 5′- GGGGTGTTGAAGGTCTCAAA-3′.

### Western blot analysis

Total proteins were extracted from cultured cells by RIPA buffer (Beyotime Institute of Biotechnology, Nantong, China) with protease inhibitors (Protease inhibitor cocktail; Roche, Indianapolis, IN). Protein concentration was determined by using BCA protein assay (Thermo Fisher Scientific, U.S.A.). The protein samples (30 μg) were separated by SDS/PAGE and transferred to polyvinylidene difluoride (PVDF) membranes (Millipore, Bedford, U.K.). The membranes were blocked with 5% nonfat milk/0.1% Tween 20 in phosphate-buffered saline (PBS-T) for 1 h at room temperature. Then the membranes were incubated with primary antibodies diluted appropriately in PBS-T at 4°C overnight. Antibodies specific to VE-cadherin (ab33168, 1:2000), VEGF (ab150766, 1:2000), β-catenin (ab32572, 1:2000), matrix metalloproteinase (MMP) 2 (MMP2) (ab97779, 1:2000), MMP9 (ab38898, 1:2000) and GAPDH (ab8245, 1:2000) were supplied by Abcam (Cambridge, U.K.). GAPDH was used as a loading control. After the incubation of primary antibodies, the membranes were washed with PBS-T for three times, 5 min each. Then the membranes were incubated with HRP–conjugated secondary antibodies (Cell Signaling Technology, Boston, U.S.A., 1:5000 in 5% nonfat milk/PBS-T) at room temperature for 1 h. After washing, the protein signals were visualized by ECL detection reagent (Amersham Biosciences, Castle Hill, Australia).

### Statistical analysis

The statistical analysis was performed with SPSS version 16.0 (IBM Chicago, IL, U.S.A.). The data are presented as the means ± SD of at least three separate experiments. The difference between two groups was analyzed by unpaired two-tailed Student’s *t* test. One-way ANOVA was used for comparison among multiple groups and multiple comparisons were further performed using post hoc Tukey test. Differences were considered statistically significant when *P*<0.05.

## Results

### HG promotes the expression of MALAT1 and VE-cadherin, and inhibits the miR-125b expression

**Figure 1:**
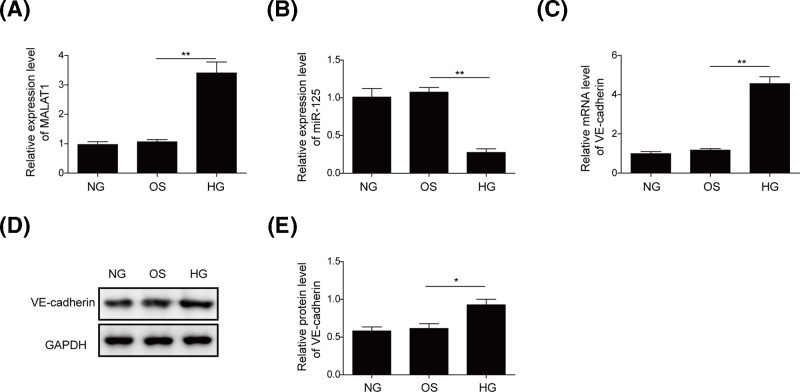
HG induced the expression of MALAT1 and VE-cadherin, and inhibited the miR-125b expression The expression level of MALAT1 (**A**) miR-125b (**B**) and VE-cadherin (**C**) was measured by qRT-PCR in hRMECs treated with HG or mannitol (high osmotic pressure, OS group), respectively. (**D**) Western blotting was used to detect VE-cadherin protein expression in hRMECs treated with HG or mannitol. GAPDH served as the loading control. (**E**) The quantitative analysis on VE-cadherin by ImageJ software. All the results were shown as mean ± SD (*n*=3), which were three separate experiments performed in triplicate. *P<0.05; **P<0.01.

To explore the effects of HG on the expression of MALAT1 and miR-125b, we used qRT-PCR and Western blotting in hRMECs exposure of HG (25 mM). qRT-PCR analysis revealed that the levels of MALAT1 and VE-cadherin were significantly increased after HG treatment (Figure 1A,C), while the level of miR-125b declined sharply (Figure 1B). Furthermore, there was no significant difference in the expression levels of MALAT1, miR-125b and VE-cadherin in the OS group compared with the NC group, suggesting that high osmotic pressure was not required for the changes of MALAT1, VE-cadherin and miR-125b expression (Figure 1A–C). We further analyzed the VE-cadherin protein expression in hRMECs with HG culture using Western blotting. As shown in Figure 1D,E, HG triggered the up-regulation of VE-cadherin protein, while high osmotic pressure had no significant effect on the protein level of VE-cadherin. Taken together, these results revealed that HG could up-regulate MALAT1 and VE-cadherin expression, simultaneously down-regulate miR-125b expression.

### MALAT1 promotes the expression of VE-cadherin via sponging miR-125b

**Figure 2:**
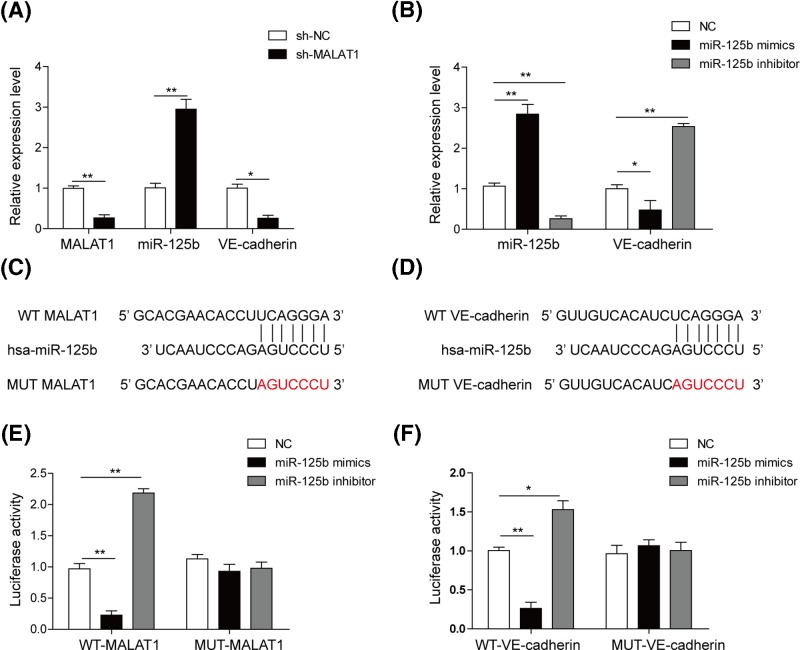
MALAT1 bound to miR-125b against VE-cadherin competitively (**A**) The expression level of MALAT, miR-125b and VE-cadherin was detected by qRT-PCR in hRMECs transfected with shMALAT1. (**B**) The expression level of miR-125b and VE-cadherin was detected by qRT-PCR in response to miR-125b mimics or miR-125b inhibitor. (**C**) Alignment of potential miR-125b binding sites in MALAT1. (**D**) Alignment of potential miR-125b binding sites in the 3′-UTR of VE-cadherin. (**E**) Luciferase activity was detected in hRMECs transfected with constructs containing wild-type of MALAT1 or mutated MALAT1 plasmid in response to the transfection of miR-125b mimics or miR-125b inhibitor. (**F**) Luciferase activity was detected in hRMECs transfected with constructs containing wild-type VE-cadherin or mutated VE-cadherin plasmid in response to the transfection of miR-125b mimics or miR-125b inhibitor. All the results were shown as mean ± SD (*n*=3), which were three separate experiments performed in triplicate. *P<0.05; **P<0.01.

We then examined the regulatory mechanisms of MALAT1, miR-125b and VE-cadherin in DR. First, to examine the effects of MALAT1 on miR-125b and VE-cadherin, we silenced MALAT1 by shMALAT1 transfection into hRMECs. The expression of MALAT1 and VE-cadherin was decreased in hRMECs transfected with shMALAT1, and miR-125b was significantly up-regulated (Figure 2A). This suggested that MALAT1 might positively regulate the expression of VE-cadherin and negatively regulate miR-125b. As shown in Figure 2B, mimics triggered miR-125b level augment and the inhibitor suppressed the miR-125b expression sharply while the results showed opposite change of VE-cadherin mRNA expressions.

Next, we applied bioinformatics methods to predict the miR-125b binding sites in MALAT1 and VE-cadherin 3′-UTR. To our surprise, we found a potential binding site in MALAT1 and VE-cadherin 3′-UTR (Figure 2C,D). To verify this result, we used dual luciferase reporting assay to test the interaction between miR-125b and the predicted MALAT1 and VE-cadherin 3′-UTR targeting sequences. It was notable that both MALAT1 and VE-cadherin 3′-UTR showed higher luciferase activities in the presence of miR-125b inhibitor while lower luciferase activities in the presence of miR-125b mimics, by contrast, the binding site mutants of MALAT1 and VE-cadherin 3′-UTR failed to display differences with the control group (Figure 2E,F). Therefore, these findings suggested that MALAT1 could directly sponge miR-125b to counteract its suppression on VE-cadherin.

### Knockdown of MALAT1 inhibits the proliferation and migration of hRMECs induced by HG through increasing miR-125b

**Figure 3:**
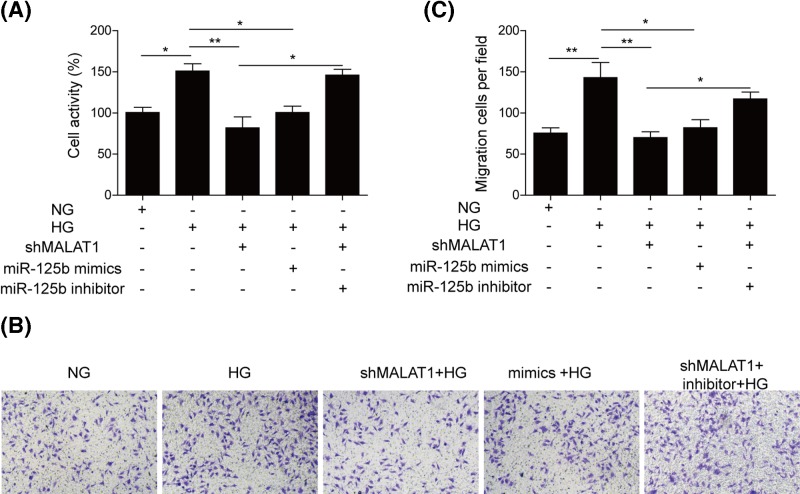
Knockdown of MALAT1 inhibited the proliferation and migration of hRMECs induced by HG through increasing miR-125b (**A**) Cell viability was detected by using MTT assay in hRMECs transfected with shMALAT1, miR-125b mimics or inhibitor. (**B**) Cell migration was assessed using transwell assay. Images of lower surface of membrane were taken after treatment with shMALAT1, miR-125b mimics or inhibitor in response to HG. Representative images were shown. (**C**) Migration was estimated by measurement of cell numbers on the lower surface of membrane. All the results were shown as mean ± SD (*n*=3), which were three separate experiments performed in triplicate. *P<0.05; **P<0.01.

The pathological changes in DR are usually characterized by abnormal proliferation and migration of retina endothelial cells [18]. To investigate the functional relevance of HG-induced MALAT1 up-regulation, we detected the effect of MALAT1 depletion on hRMECs viability and migration. As shown in Figure 3A, MTT assay revealed that HG promoted the cell activity, whereas both the deletion of MALAT1 and the miR-125 mimics could inhibit HG-induced cell proliferation outstandingly. However, miR-125b inhibitor rescued the declined cell viability induced by shMALAT1 transfection (Figure 3A). The results of transwell assay demonstrated that knockdown of MALAT1 and overexpression of miR-125b could repress cell migration of hRMECs, while miR-125b inhibitor could reverse migration inhibition induced by shMALAT1 (Figure 3B,C). Collectively, these results suggested that silence of MALAT1 inhibited the proliferation and migration of hRMECs induced by HG through up-regulating miR-125b.

### Knockdown of MALAT1 suppresses tube formation and vascular permeability of hRMECs induced by HG via up-regulating miR-125b

**Figure 4:**
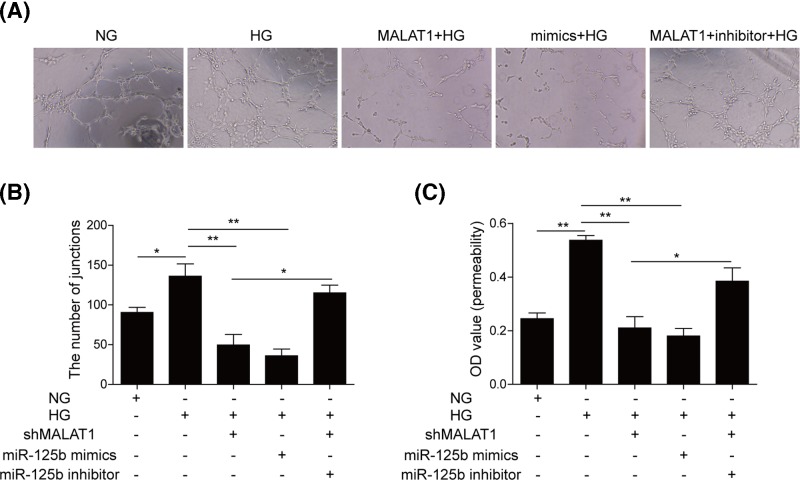
Knockdown of MALAT1 inhibited the tube formation and vascular permeability of hRMECs induced by HG through targeting miR-125b (**A**) Tube formation assay was used to detect tube-formation capacity of hRMECs transfected with shMALAT1, miR-125b mimics or inhibitor. (**B**) The average number of tube formation for each field was statistically analyzed. (**C**) The average OD value of permeability was calculated and shown. All the results were shown as mean ± SD (*n*=3), which were three separate experiments performed in triplicate. *P<0.05; **P<0.01.

To investigate the role of MALAT1 in the angiogenesis, we measured the effects of MALAT1 and miR-125b on tube formation capacity and vascular permeability of hRMECs in the presence of HG. The ability to form a tubular network of hRMECs was evaluated using tube formation assay. HG treatment led to a significant increase in the number of capillary-like structure, whereas MALAT1 knockdown and miR-125b mimics significantly decreased the number (Figure 4A,B). By contrast, miR-125b inhibitor reversed shMALAT1-mediated inhibition of endothelial capillary tube formation (Figure 4B). Additionally, miR-125b mimics and MALAT1 silence inhibited the vascular permeability, which was rescued by miR-125b inhibitor (Figure 4C). Collectively, these data revealed that knockdown of MALAT1 could suppress the tube formation and vascular permeability of hRMECs induced by HG through targetig miR-125b.

### Knockdown of MALAT1 suppresses the VE-cadherin/β-catenin complex and neovascularization related proteins expression induced by HG

**Figure 5:**
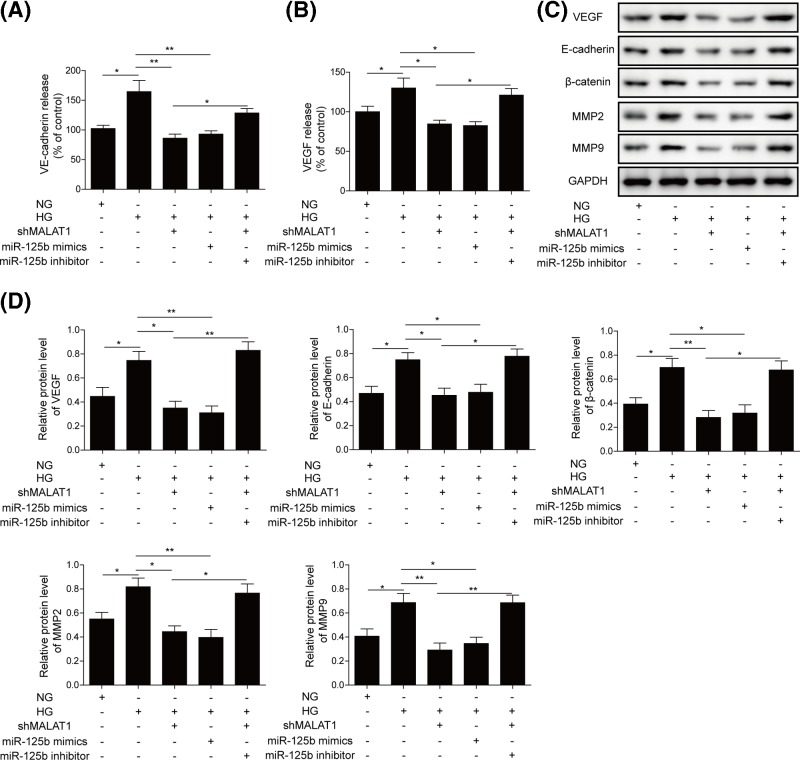
Knockdown of MALAT1 suppressed the VE-cadherin/β-catenin complex and neovascularization-related proteins expression activated by HG The release level of VE-cadherin (**A**) and VEGF (**B**) was decreased by ELISA assay in hRMECs transfected with shMALAT1, miR-125b mimics or inhibitor. (**C**) The protein expression of VEGF, VE-cadherin, β-catenin, MMP2 and MMP9 were examined by Western blotting in hRMECs transfected with shMALAT1, miR-125b mimics or inhibitor. GAPDH served as the loading control. (**D**) The quantitative analysis on VEGF, VE-cadherin, β-catenin, MMP2 and MMP9 was carried out by ImageJ software. All the results were shown as mean ± SD (*n*=3), which were three separate experiments performed in triplicate. *P<0.05; **P<0.01.

VE-cadherin/β-catenin complex is the backbone of adherent type junctions in endothelium. We explored the effect of MALAT1 on the E-cadherin/β-catenin complex and neovascularization related proteins’ expression. As shown in Figure 5A,B, HG enhanced VE-cadherin and VEGF release, while shMALAT1 and miR-125b mimics transfection outstandingly reduced the release of VE-cadherin and VEGF in hRMECs. However, miR-125b inhibitor repressed the decline of VE-cadherin and VEGF release mediated by shMALAT1 transfection (Figure 5A,B). Furthermore, we found that the MALAT1 deletion inhibited neovascularization related proteins expression, including VEGF, VE-cadherin, β-catenin, MMP2 and MMP9, which was then rescued by the miR-125b inhibitor (Figure 5C,D). These data clearly suggested that the deletion of MALAT1 suppressed the release of VE-cadherin and VEGF by inhibiting VE-cadherin/β-catenin complex by targeting miR-125b, which alleviated neovascularization induced by HG.

## Discussion

DR is a chronic and serious eye complication associated with DM, and people with DR present an increased risk of other microvascular and macrovascular complications associated with diabetes. Hyperglycemia is closely related to the pathologic changes related to DR, which are responsible for induction of neovascularization, inflammation, oxidative stress, apoptosis and cell proliferation. There is no effective cure for DR so far, and its underlying molecular mechanism is not yet fully understood. The purpose of the present study was to investigate the role of MALAT1 in DR and its potential molecular regulatory mechanisms. Our study revealed that the expression of MALAT1 and VE-cadherin was triggered by HG and the expression of miR-125b was inhibited by HG. Additionally, MALAT1 bound to miR-125b competitively against VE-cadherin resulted in VE-cadherin activation. Knockdown of MALAT1 inhibited the proliferation, migration, tube formation and vascular permeability of hRMECs induced by HG through restraining VE-cadherin/β-catenin complex by targeting miR-125b.

MALAT1, as an evolutionarily conserved lncRNA, was reported to be correlated with angiogenesis. Zhang et al. [19] demonstrated that knockdown of MALAT1 could significantly decrease tube formation, cell migration and cell proliferation in mouse primary skeletal muscle microvascular endothelial cells. The silence of MALAT1 could suppress cell motility, proliferation and increased apoptosis in bladder cancer cells [20]. Furthermore, MALAT1 could promote angiogenesis of thyroid cancer by regulating tumor-associated macrophage FGF2 protein secretion [21]. MALAT1 was significantly up-regulated in DM and DR [11,22]. Zhang et al. [22] reported that the expression level of MALAT1 was higher in the gestational DM patients group than in the normal group. Liu et al. [11] revealed that MALAT1 level was significantly up-regulated in diabetic animal models, and MALAT1 knockdown ameliorated retinal function and retinal vessel impairment in diabetic rats. This implied that MALAT1 might be a key regulator of DR, but the underlying mechanism of MALAT1 was still unclear in DR. In the present study, we found that MALAT1 was significantly increased in hRMECs treatment with HG. Knockdown of MALAT1 inhibited the cell viability, migration, tube formation and vascular permeability of hRMECs induced by HG via directly inhibiting miR-125b, which meant MALAT1 might be a new target for DR therapy, and miR-125b mimic might provide a promising therapeutic strategy for DR patients.

By binding to specific sites within the 3′-UTR, miRNAs can reduce gene expression of various mRNAs by inhibiting translation or directly causing transcript degradation. miR-125b was reported to bind directly to the 3′-UTR of VE-cadherin and inhibit its translation [16]. Moreover, miR-125b could inhibit tube formation of blood vessels through suppressing VE-cadherin [16]. Furthermore, Gong et al. [14] identified lowly expressed miR-125b associated with cellular dysfunction in early DR. In this study, we revealed that miR-125b was down-regulated in response to HG. Furthermore, miR-125b could be bound by MALAT1 against VE-cadherin at the region of 3′-UTR, leading to the declined down-regulation of VE-cadherin. Our study demonstrated the underlying mechanism of MALAT1 in DR.

As a type-II endothelial-restricted cadherin, VE-cadherin is the major transmembrane adhesion molecule of endothelial adherens junctions [23]. Previous research illuminated that VE-cadherin could be involved in regulation of vascular diseases [5,24]. Substantial evidence has strengthened that the adhesiveness of VE-cadherin was correlated to the tyrosine phosphorylation of the VE-cadherin/catenin complex [25]. VE-cadherin gene silence results in severe angiogenic defects, leading to abnormal VEGF signaling [26]. VEGF recently is thought to be an important angiogenesis factor in DR [27]. In addition, there is evidence to suggest that pericyte support of vessel survival is closely related to the expression of VEGF [28]. Emerging evidence revealed VEGF-antibody could be novel therapy for treating DR [29]. Besides, angiogenesis factors included MMP2 and MMP9, which were downstream of VEGF and activated by VEGF [30]. In the present study, we found the deletion of MALAT1 suppressed the VE-cadherin/β-catenin complex and neovascularization related proteins VEGF, MMP2 and MMP9 expression activated by HG.

In conclusion, the present study revealed that MALAT1 bound to miR-125b against VE-cadherin competitively at the site of 3′-UTR. Knockdown of MALAT1 restrained cell migration, tube formation and vascular permeability of hRMECs induced by HG through directly up-regulating miR-125b. Furthermore, the silence of MALAT1 inhibited VEGF secretion and neovascularization related proteins expression via repressing VE-cadherin/β-catenin complex. The present study provides novel insights into MALAT1 in DR and may help improve the development of DR therapy.

## Abbreviations

BMM: basement membrane matrix
DM: diabetes mellitus
DR: diabetic retinopathy
ELISA: enzyme linked immunosorbent assay
FBS: fetal bovine serum
FGF2: fibroblast growth factor-2
GAPDH: glyceraldehyde-3-phosphate dehydrogenase
HG: high glucose
hRMEC: human retina microvascular endothelial cell
HRP: horse radish peroxidase
lncRNA: long non-coding RNA
MALAT1: metastasis associated lung adenocarcinoma transcript 1
miRNA: microRNA
MMP: matrix metalloproteinase
MTT: 3-(4,5-dimethylthiazol-2-yl)-2,5-diphenyltetrazolium bromide
PBS-T: 0.1% Tween 20 in phosphate-buffered saline
qRT-PCR: quantitative real-time PCR
VE-cadherin: vascular endothelial-cadherin
VEGF: vascular endothelial growth factor
3′-UTR: 3′-untranslated region
